# Has growth in electronic cigarette use by smokers been responsible for the decline in use of licensed nicotine products? Findings from repeated cross-sectional surveys

**DOI:** 10.1136/thoraxjnl-2015-206801

**Published:** 2015-07-24

**Authors:** Emma Beard, Jamie Brown, Ann McNeill, Susan Michie, Robert West

**Affiliations:** 1 Cancer Research UK Health Behaviour Research Centre, University College London, London, UK; 2 Research Department of Educational, Clinical and Health Psychology, University College London, London, UK; 3 Addictions Department, Institute of Psychiatry, King's College London, London, UK

**Keywords:** Tobacco and the lung

## Abstract

**Background:**

The rise in electronic cigarette use by smokers may be responsible for the decreased use of licensed nicotine products and/or increased overall use of non-tobacco nicotine-containing products. This paper reports findings from the Smoking Toolkit Study (STS) tracking use of electronic cigarettes and licensed nicotine products to address this issue.

**Methods:**

Data were obtained from monthly surveys involving 14 502 cigarette smokers in England between March 2011 and November 2014. Smokers were asked about their use of electronic cigarettes and licensed nicotine products.

**Results:**

Prevalence of electronic cigarette use increased rapidly from 2.2% (95% CI 1.4% to 3.2%) in quarter 2 of 2011 to 20.8% (95% CI 18.3% to 23.4%) in quarter 3 of 2013, after which there was no change. Prevalence of licensed nicotine product use in smokers remained stable from quarter 2 of 2011 (17.4%, 95% CI 15.3% to 19.8%) to quarter 3 of 2013 (17.9%, 95% CI 15.62% to 20.5%), and thereafter declined steadily to 7.9% (95% CI 6.0% to 10.4%). Prevalence of use of any product was stable to quarter 1 of 2012, after which it increased from 18.5% (95% CI 16.3% to 21.0%) to 33.3% (95% CI 30.4% to 36.3%) in quarter 3 of 2013, and then decreased to 22.7% (95% CI 19.3% to 26.3%).

**Conclusions:**

The shapes of trajectories since 2011 suggest that electronic cigarettes are probably not responsible for the decline in use of licensed nicotine products. Electronic cigarettes appear to have increased the total market for use of non-tobacco nicotine-containing products.

**Key messages**

**What is the key question?**

- Has the rapid rise in the use of electronic cigarettes by smokers led to the decline in the use of licensed nicotine products?

**What is the bottom line?**

- The shapes of the trajectories of the use of electronic cigarettes and licensed nicotine products suggest that growth in the use of electronic cigarettes is probably not responsible for the decline in licensed nicotine product use by smokers but instead appears to have increased the market for non-tobacco nicotine-containing products.

**Why read on?**

- This study provides the first evidence in any country assessing the potential trade-off between the use of electronic cigarettes and nicotine products licensed by medicines regulators.

## Introduction

Electronic cigarettes are battery-powered devices that can provide inhaled doses of nicotine by way of a vaporised solution. Since they were introduced to the European market in 2006, there has been substantial growth in their use by smokers.^1^ Supermarket sales data showed a rise of over 40% in their use between 2013 and 2014.^2^ In contrast, licensed nicotine products appear to have become less popular, with sales in many European countries having decreased over the past few years (eg, France and the UK).^3^ ^4^

The use of licensed nicotine products for smoking reduction appears to promote quit attempts.^5^ Thus if electronic cigarette use is substituting for licensed nicotine product use among smokers, and does not promote cessation, there could be a negative net effect on public health. On the other hand, if electronic cigarette users are primarily smokers who would have tried to reduce without any nicotine product use or not tried to reduce at all, there may be a public health gain, as long as electronic cigarette use also promotes subsequent cessation. Although electronic cigarettes are almost certainly considerably safer than traditional cigarettes, and when used for cessation they probably improve the chances of success,^1^ ^6^ ^7^ evidence on the benefits or otherwise of electronic cigarette use while continuing to smoke is mixed.^1^ ^6–11^

It is therefore important to determine how far electronic cigarette use has replaced or supplemented licensed nicotine product use. The introduction of new smoking cessation aids on to the market has previously resulted in more smokers using medications of some sort to help them stop.^12^ ^13^ However, this may not be the case for electronic cigarettes and smoking reduction. To address the issue of how far electronic cigarettes are complementing licensed nicotine products or replacing them, one can assess whether the temporal trajectories in prevalence of use of the two types of product mirror each other. If they follow very different patterns of change, then it is unlikely that they are connected. Therefore, this study set out to answer the following questions:

1. What has been the trajectory in growth of electronic cigarette use in current smokers, and how has this compared with the trajectory in decline in use of licensed nicotine products?
2. What has been the resultant trajectory in use of any nicotine-containing product?

## Methods

### Design

The study formed part of the Smoking Toolkit Study (STS), an ongoing population study designed to provide information on smoking and smoking cessation patterns among smokers and recent ex-smokers in England. Data for this paper were obtained between March 2011 and August 2014. The STS involves monthly household surveys using a random location sampling design, with initial random selection of grouped output areas (containing 300 households), stratified by ACORN (socio-demographic) characteristics http://acorn.caci.co.uk/ and region. Interviewers then choose which houses within these areas are most likely to fulfil their quotas and conduct face-to-face computer-assisted interviews with one member per household.^14^ Participants in the STS appear to be representative of the population in England, having a similar socio-demographic composition to other large national surveys, such as the Health Survey for England.^14^

### Measures

Smokers were asked:

1. ‘Are you using any of the following either to help you stop smoking, to help you cut down or for any other reason at all?’ Answer: nicotine patch; nicotine gum; nicotine lozenges/tablets; nicotine inhaler; nicotine nasal spray; mouth spray; electronic cigarettes; I don't know; none of these; other

Smokers were also asked if they were attempting to cut down their cigarette consumption and:

1. ‘Which, if any, of the following are you currently using to help you cut down the amount you smoke?’ Answer: nicotine patch; nicotine gum; nicotine lozenges/tablets; nicotine inhaler; nicotine nasal spray; mouth spray; electronic cigarettes; I don't know; none of these; other
2. ‘Do you regularly use any of the following in situations when you are not allowed to smoke?’ Answer: nicotine patch; nicotine gum; nicotine lozenges/tablets; nicotine inhaler; nicotine nasal spray; mouth spray; electronic cigarettes; I don't know; none of these; other

Respondents were classified accordingly:

- Using electronic cigarettes: reported using electronic cigarettes in response to question 1 and/or 2 and/or 3
- Using licensed nicotine products: reported using any of the licensed products in response to question 1 and/or 2 and/or 3
- Using nicotine-containing products: reported using licensed nicotine products and/or e-cigarettes in response to question 1 and/or 2 and/or 3.

Contextual information was also gathered on socio-demographic and smoking-related characteristics (ie, gender, age, socio-economic status, cigarette consumption, cigarette dependence, daily versus non-daily nicotine-containing product use, attempts to quit smoking in the previous 12 months and attempts to cut down cigarette consumption). Socio-economic status was measured using the Social-Grade Classification Tool,^15^ which categorises individuals into one of five social grades: AB, C1, C2, D and E. Grades AB and C1 were classified as ‘non-manual’ and Grades C2 to E were classified as ‘manual’ occupational groups. Cigarette dependence was assessed using time to first cigarette of the day.^16^ A copy of the questionnaire is available on the STS website (http://www.smokinginengland.info)

### Statistical analysis

Analyses were undertaken using R V.3.1.1. Data were weighted to match the population in England (see Fidler *et al*^14^ for details). Differences in socio-demographic and smoking characteristics as a function of nicotine-containing product use were assessed with generalised linear models (for normally distributed outcomes) and χ^2^ tests (for dichotomous outcomes), using the ‘Survey’ R package.^17^ Post hoc analyses were conducted using multiple χ^2^ and t tests, and were adjusted using the Benjamini and Yekutieli false discovery rate.

Data were aggregated into quarters to reduce the sampling variation associated with each data point. Trends in prevalence of use of electronic cigarettes, licensed nicotine products and nicotine-containing products were first assessed using generalised linear models (specifying the binomial family and logit link function). However, as it was hypothesised that the trends may be inconsistent over time (eg, there may be an initial increase then decrease in prevalence or vice versa), segmented regression models were also applied. These are regression models where the relationship between the outcome and the predictor variables are *piecewise linear*, namely represented by at least two straight lines connected at ‘breakpoints’. For this paper, a breakpoint would occur when there was a change in the slope of the function relating prevalence to time. Segmented regressions were applied using the ‘segmented’ package,^18^ which allows both single and multiple breakpoints to be specified. This programme uses an iterative procedure whereby only starting values for the breakpoints are required. It also implements bootstrap restarting to make the algorithm less sensitive to starting values.

To determine whether a model with breakpoints provided a better fit than a standard generalised linear model, the Davies test assessed the null hypothesis that there was no difference in slopes before and after a breakpoint. In all cases, the difference in slopes was significantly greater than 0. As recommended for segmented regression analyses, the Bayesian Information Criterion was used to select the optimal number of breakpoints (ie, 1, 2 or 3).^19^

Because time is a predictor in the regression analyses, error terms of consecutive observations may be correlated. This may lead to underestimation of SEs and overestimation of statistical significance.^20^ The presence of correlations between consecutive time points was assessed using the Durbin-Watson statistic. Based on a critical value of 2.0, there was no evidence of significant autocorrelation (values ranged from 1.91 to 1.97).

There were missing data for four of the socio-demographic and smoking characteristic variables. For frequency of licensed nicotine product and/or electronic cigarette use, missing data ranged from 7% among electronic cigarette users to 20% among those using both products, while less than 3% of participants had missing data on age, previous attempts to quit smoking and attempts to cut down. Missing data were imputed using the ‘Amelia 11’ package.^21^ The number of imputed data sets was based on the recommendations of Graham *et al*^22^ and set to 10. Estimates and results from hypothesis testing were combined using Rubin's rules.^23^ STROBE guidelines for the reporting of observational studies were followed.^24^

## Results

Between March 2011 and November 2014 data were collected from 14 502 cigarette smokers. Overall, 76% (95% CI 75.3% to 76.7%) were not using any form of nicotine-containing product, 9.9% (95% CI 9.4% to 10.4%) were using electronic cigarettes, 11.6% (95% CI 11.1% to 12.2%) were using licensed nicotine products, and 2.4% (95% CI 2.2% to 2.7%) were using both of these types of product.

Table 1 shows the characteristics of participants as a function of their nicotine-containing product use. Those not using nicotine-containing products were less likely to be female compared with those using licensed nicotine products (OR 0.81; χ^2^=17.23, df=1, p=0.003) and were more likely to be in a manual job than those using electronic cigarettes (OR=1.30; χ^2^=21.80, df=1, p<0.001) and those using licensed nicotine products (OR=1.18; χ^2^=10.27, df=1, p=0.035). Those using licensed nicotine products were older than those not using any nicotine-containing product (mean difference 0.94; t(12 835)=4.35, p<0.001) and those using electronic cigarettes (mean difference 2.21; t(2998)=2.72, p=0.003).

**Table 1:** Characteristics of smokers as a function of their use of nicotine-containing products

	Not using licensed nicotine products or electronic cigarettes	Using electronic cigarettes only	Using licensed nicotine products only	Using electronic cigarettes and licensed nicotine products
	N=10 431	N=1360	N=1603	N=330
Female % (n)	44.7 (4667)^a^	48.8 (664)	50.1 (803)^b^	51.2 (169)
Age mean (SD)	41.5 (17.56)^b^	41.2 (16.13)^b^	43.3 (16.17)^a^	42.5 (15.76)
Manual occupation % (n)	61.7 (6434)^a^	55.4 (752)^b^	57.6 (924)^b^	54.7 (180)
Time to first cigarette mean (SD)	1.3 (1.16)^a^	1.4 (1.15)^b,f^	1.5 (1.17)^b,f^	1.7 (1.11)^b,e^
Attempts to quit smoking in the previous 12 months % (n)	23.4 (2452)^a^	53.7 (729)^b^	63.2 (1013)^c^	72.1 (237)^c^
Non-daily smoking % (n)	10.3 (1077)	11.8 (160)	10.7 (171)	11.6 (38)
Attempting to cut down % (n)	41.7 (4349)^a^	76.1 (1034)^b^	77.9 (1248)^b^	78.5 (259)^b^
Using nicotine-containing products for				
Smoking reduction % (n)	NA	69.2 (942)	70.5 (1130)	74.8 (247)
Temporary abstinence % (n)	NA	71.4 (971)^a^	64.4 (1032)^b,e^	78.9 (260)^b,f^
Daily nicotine-containing product use % (n)	NA	56.8 (772)^a^	52.4 (841)	47.1 (155)^b^

Data are weighted to match the English population (weighted n's are presented); n's were rounded up to the nearest whole number. As a consequence percentages may not sum to 100 and n's may not sum to total N. Social grade was measured using the social grades system: non-manual includes A: higher managerial, administrative or professional, B: intermediate managerial, administrative or professional, C1: supervisory or clerical and junior managerial administrative or professional; manual includes C2: skilled manual workers, D: semi and unskilled manual workers, E: casual or lowest grade workers, pensioners and others who depend on the welfare state for their income. AB and C1 were coded as non-manual and C2, D and E as manual.^17^ Time to first cigarette of the day was assessed as (1) >60 min, (2) 31–60 min, (3) 6–30 min or (4) ≤5 min. a, b, c and d all differ; e differs from f. NA, not applicable.

Those not using any nicotine-containing product had lower odds of reporting that they were attempting to cut down compared with those using electronic cigarettes (OR 0.27; χ^2^=607.12, df=1, p<0.001), those using licensed nicotine products (OR 0.18; χ^2^=762.93, df=1, p<0.001) and those using both products (OR 0.12; χ^2^=231.23, df=1, p<0.001). Those using licensed nicotine products had higher odds than those using electronic cigarettes (OR 1.48; χ^2^=28.22, df=1, p<0.001) of having made a quit attempt and electronic cigarette users had higher odds than those not using any product (OR 3.78; χ^2^=587.86, df=1, p<0.001). Those using both products were more cigarette dependent than those using only licensed nicotine products or electronic cigarettes (mean difference 0.11; t(1663)=3.29, p=0.020 and mean difference 0.18; t(1975)=2.73, p=0.005, respectively), while those not using any nicotine-containing product had lower cigarette dependence compared with those using electronic cigarettes, those using licensed nicotine products and those using both products (mean difference 0.09; t(12 523)=2.71, p=0.020, mean difference 0.07; t(12 834)=4.19, p<0.001, and mean difference 0.11; t(11 500)=5.08, p<0.001, respectively). There was no difference among groups in the percentage reporting non-daily smoking (χ^2^=3.22, df=3, p=0.463). Of those using some form of nicotine-containing product, those using both electronic cigarettes and licensed nicotine products were less likely to report daily use of electronic cigarettes and/or licensed nicotine products compared with those using electronic cigarettes only (OR 2.22; χ^2^=9.75, df=1, p=0.020). Those using both products also had higher odds of reporting that they were doing so during temporary abstinence relative to electronic cigarette users (OR 1.50; χ^2^=7.38, df=1, p=0.026), who had a higher odds of reporting they were using electronic cigarettes for temporary abstinence compared to those using licensed nicotine products only (OR 1.38; χ^2^=16.81, df=1, p<0.001). There was no difference among groups in reports of using nicotine-containing products and/or electronic cigarettes to reduce cigarette consumption (χ^2^=4.05, df=2, p=0.180).

**Figure 1:**
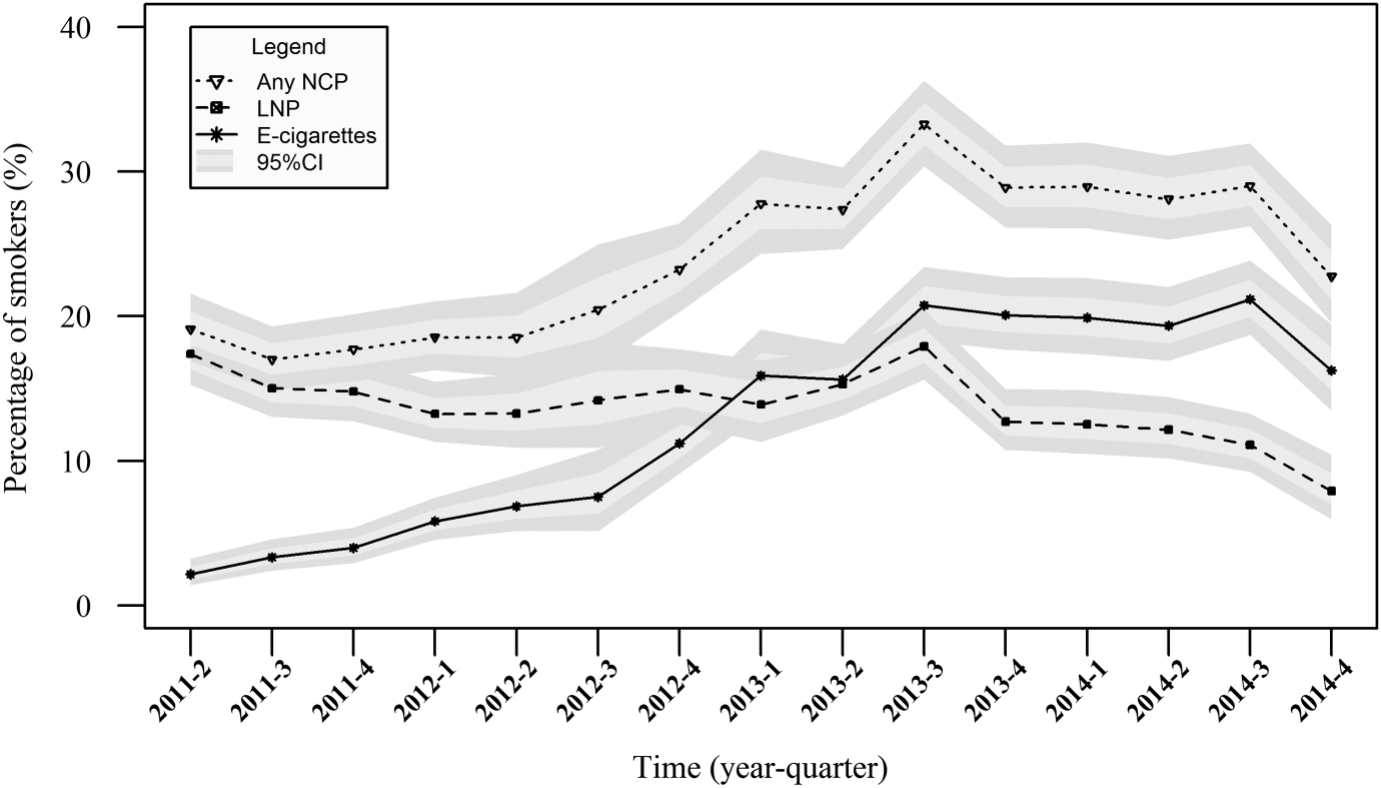
The proportion of smokers using nicotine-containing products over time. Data weighted to match the English population. E-cigarettes, electronic cigarettes; LNP, licensed nicotine product; NCP, nicotine-containing product. Time is represented quarterly in years.

**Figure 2:**
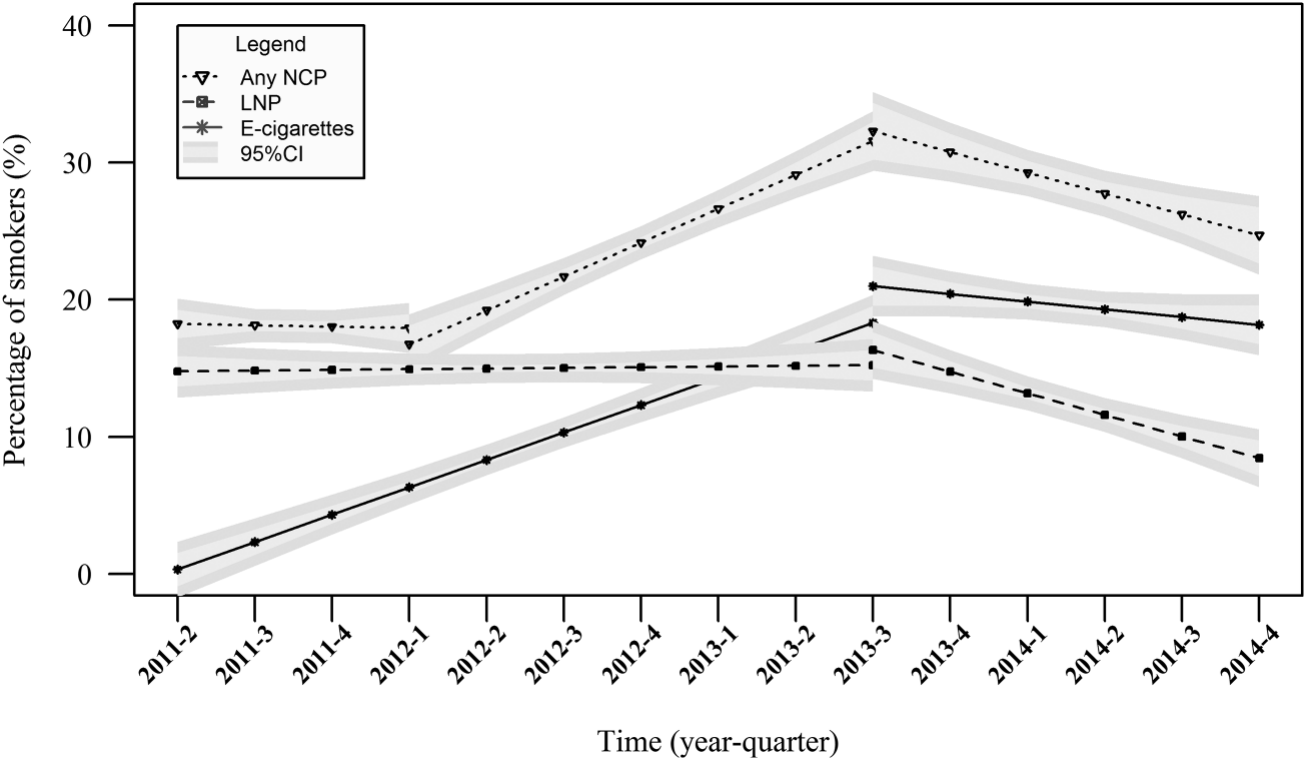
The proportion of smokers using nicotine-containing products over time: segmented regression results. Data weighted to match the English population; E-cigarettes, electronic cigarettes; LNP, licensed nicotine product; NCP, nicotine-containing product. Time represented quarterly in years.

Figure 1 shows the prevalence of electronic cigarette, licensed nicotine product and nicotine-containing product use, while figure 2 displays the results of the segmented regression analyses. The segmented regression analyses showed that there was a rapid increase in electronic cigarette use up to quarter 3 of 2013 from 2.2% (95% CI 1.4% to 3.2%) to 20.8% (95% CI 18.3% to 23.4%) (OR 1.31, 95% CI 1.28% to 1.35%, p<0.001) and little change thereafter (OR 0.96, 95% CI 0.91% to 1.01%, p=0.129) with a prevalence in quarter 4 of 2014 of 16.3% (95% CI 13.5% to 19.5%). In contrast, there was no change in licensed nicotine product use between quarter 2 of 2011 (17.4%, 95% CI 15.3% to 19.8%) and quarter 3 of 2013 (17.9%, 95% CI 15.62% to 20.5%; OR 1.01, 95% CI 0.99 to 1.03, p=0.277); and there was a continual slow decline in licensed nicotine product use thereafter (OR 0.90, 95% CI 0.84 to 0.96, p=0.003). The prevalence in quarter 4 of 2014 was 7.9% (95% CI 6.0% to 10.4%).

The result was that there was no increase in nicotine-containing product use between quarter 2 of 2011 (19.1%, 95% CI 16.9% to 21.5%) and quarter 1 of 2012 (18.5%, 95% CI 16.3% to 21.0%; OR 0.97, 95% CI 0.91% to 1.04%, p=0.297), an increase to 33.3% (95% CI 30.4% to 36.3%) up to quarter 3 of 2013 (OR 1.16, 95% CI 1.12 to 1.21, p<0.001) and then a decrease to 22.7% (95% CI 19.3% to 26.3%) in quarter 4 of 2014 (OR 0.94, 95% CI 0.90 to 0.99, p=0.014).

## Discussion

There was a rapid increase in electronic cigarette use by smokers between quarter 2 of 2011 and quarter 3 of 2013 with little change thereafter. Over the same period, licensed nicotine product use remained stable and then dropped gradually between quarter 3 of 2013 and quarter 4 of 2014. The result was an initial growth in nicotine-containing product use up to quarter 3 of 2013 and a decrease thereafter. These trajectories suggest that electronic cigarette use is not associated with the reduction in licensed nicotine product use by smokers but may have instead increased the market for nicotine-containing products.

If the rise in electronic cigarette use has not been primarily responsible for the decline in the use of licensed nicotine products by smokers, this raises the question as to what has caused this decrease. There was no reduction in the percentage of smokers attempting to reduce their smoking, so that is unlikely to explain the trend.^25^ The stop smoking services recommend licensed nicotine products and offer them on prescription and use of these services has declined since 2011.^26^ However, this decline is unlikely to be an important factor as the large majority of smokers who previously used licensed nicotine products while smoking had not attended stop smoking services.^27^ Marketing of licensed nicotine products increased over the period of data collection, so reduced exposure to advertising does not appear to be a factor.^28^ It is possible that the trend reflects a longer term disillusionment with licensed nicotine products as aids to smoking reduction. This is something that warrants further investigation.

In terms of what may underlie the trajectory in electronic cigarette use, the rapid rise could be explained by social contagion with more and more smokers being persuaded that these products could help them reduce the amount they smoke or ultimately stop altogether. The plateau could reflect a ceiling on the proportion of smokers who want to reduce their cigarette consumption enough to buy a nicotine product, or it could be that an increasing proportion of smokers have been led to believe that electronic cigarettes are as harmful as tobacco cigarettes,^29^ thus removing the incentive to use them. This is another important area for future study.

This study has several strengths including the large number of participants, the representativeness of the sample and the use of frequent sampling. There were also a number of limitations. This study only considered electronic cigarette and licensed nicotine product use by current smokers. Use specifically as an aid to cessation and use among never and ex-smokers are important further topics for investigation. Beyond assessing daily versus non-daily use, this study did not examine the frequency of electronic cigarette and licensed nicotine product use. This study did not assess to what extent electronic cigarette or licensed nicotine product use was associated with a reduction in cigarette consumption or intake of tobacco toxins. Because electronic cigarette and licensed nicotine product users are likely to have been heavier smokers before they started using the products, this can only be assessed using prospective designs.^1^ The data series was limited to approximately 3 years. It will be important to continue to monitor the trends to track further changes as the electronic cigarette market continues to mature and the regulatory environment changes. As with all survey-based designs, there is a possibility of misreporting of nicotine-containing product use although there is no reason to believe that this varied over time.

In conclusion, the rapid growth in electronic cigarette use between 2011 and 2013 appears to have increased the overall market for use of nicotine products while smoking and was not associated with a decline in use of licensed nicotine products over this period.
